# Exercise-related anaphylaxis with food allergy to cross-reactive LTPs

**DOI:** 10.1186/2045-7022-3-S3-P162

**Published:** 2013-07-25

**Authors:** T Vieira, AM Pereira, D Silva, A Moreira, L Delgado

**Affiliations:** 1 Department of Allergy, Hospital São João, EPE, Porto, Portugal; 2 Immunology Lab, Department of Clinical Pathology, Hospital São João, EPE, Porto, Portugal; 3 Department of Immunology, University of Porto, Porto, Portugal; 4 CINTESIS and Biostatistics and Medical Informatics, Faculty of Medicine, University of Porto, Porto, Portugal

## Background

In food dependent exercise-induced anaphylaxis, a food challenge followed by an exercise test, being the gold standard for diagnosis, might not be feasible under certain circumstances. Therefore, *in vitro* diagnostic tests can be helpful to clarify severe forms of exercise-related hypersensitivity syndromes. We report two patients with food allergy to non-specific LTPs presenting as exercise-related anaphylaxis after food intake. Our aim was to identify IgE cross-reactivities using an *in-vitro* depletion assay with the suspected native allergens, and an allergen microarray.

## Methods

### Case 1

A 25-year-old man with cholinergic urticaria and mild OAS to peach, suffered an episode of generalized urticaria and angioedema during a recreational soccer match, which was preceded by peach based soft drink ingestion.

### Case 2

A 19-year-old woman with controlled asthma and allergic rhinitis to mites and grass pollens, suffered an anaphylactic shock while playing soccer. She had ingestedwalnuts 1 ½ hours before, and tomato, mango, orange and wheat bread with cheese within the previous 6 hours.

No other episodes of anaphylaxis to foods were declared in both patients.

Standard investigations included skin tests (ST) and specific IgE (sIgE). Confirmative oral challenges followed by an exercise test were precluded as case 1 suffered from cholinergic urticaria and case 2 had a life-threatening reaction.

Immunodepletion was performed with the serum of each patient using the suspected native allergen (peach and walnut, respectively) and the samples re-tested with ImmunoCAP ISAC.

## Results

In case 1, ST and ISAC confirmed IgE sensitization only to LTP components. After serum pre-incubation, there was a 100% depletion of sIgE to all cross-reactive LTPs tested in ISAC.

In case 2, ST and sIgE were positive to various fruits of *Rosaceae* and non *Rosaceae* family and nuts, including mango, orange and walnuts. ISAC detected sensitization to all LTPs tested, with species-specific sensitization to mites and grass pollens. The percentages of sIgE depletion ranged from 60% (Ara h 9) to 100% (Cor a 8).

## Conclusion

In both patients sIgE to cross-reactivity fruit LTPs was clearly demonstrated by the *in vitro* methods. Exercise may be a relevant co-factor for a severe clinical presentation in LTP-related food allergy. We stress the relevance of an accurate identification of culprit allergens in life-threatening events, when re-challenge might not be feasible.

## Disclosure of interest

None declared.

